# Application and risk prediction of thrombolytic therapy in cardio-cerebrovascular diseases: a review

**DOI:** 10.1186/s12959-023-00532-0

**Published:** 2023-09-04

**Authors:** Kexin Zhang, Yao Jiang, Hesong Zeng, Hongling Zhu

**Affiliations:** Division of Cardiology, Department of Internal Medicine, Tongji Hospital, Tongji Medical College, Huazhong University of Science and Technology, Wuhan, 430030 Hubei China

**Keywords:** Ischemic stroke, Myocardial infarction, Machine learning, Risk assessment, Thrombolytic therapy

## Abstract

Cardiocerebrovascular diseases (CVDs) are the leading cause of death worldwide, consuming huge healthcare budget. For CVD patients, the prompt assessment and appropriate administration is the crux to save life and improve prognosis. Thrombolytic therapy, as a non-invasive approach to achieve recanalization, is the basic component of CVD treatment. Still, there are risks that limits its application. The objective of this review is to give an introduction on the utilization of thrombolytic therapy in cardiocerebrovascular blockage diseases, including coronary heart disease and ischemic stroke, and to review the development in risk assessment of thrombolytic therapy, comparing the performance of traditional scales and novel artificial intelligence-based risk assessment models.

**Supplementary Information:**

The online version contains supplementary material available at 10.1186/s12959-023-00532-0.

## Introduction

Cardiocerebrovascular diseases (CVDs) are pathological conditions involving the cardiovascular system, which are the leading cause of death worldwide, and more than 80% CVD-caused deaths are due to CHD and stroke [1]. According to data from WHO, in 2019, about 17.9 million people died from CVDs, accounting for 32% of the global total. In the United States, the main type of CVDs was CHD, with a proportion of 41.3%, followed by stroke (17.2%) [2]. CHDs are the stenosis or obstruction of the coronary artery, leading to myocardial ischemia, hypoxia and even necrosis. One of the most important characteristics of CHD is atherosclerosis. (The pathogenesis of atherosclerosis is shown in Additional file 1). According to their stability, atheromatous plaques are divided into stable plaques and unstable plaques. When the unstable plaque ruptures or erodes, subendothelial collagen, lipid core, and procoagulants like tissue factor and von Willebrand factor are exposed to the blood circulation, which rapidly promotes platelets to adhere to the vessel wall and subsequently aggregate, contributing to acute thrombosis. Then the coronary artery is completely blocked, and later the ischemia and hypoxia of the myocardium in the corresponding area emerge, resulting in myocardial infarction characterized by ST-segment elevation (STEMI), which is a serious type of acute coronary syndrome (ACS).

Another fatal type of CVD, stroke, is divided into hemorrhagic stroke and ischemic stroke based on its pathogenesis. Ischemic stroke is the main type of stroke, accounting for 85% of strokes. It is defined as a result of thrombosis or embolism that blocks cerebral vessels in a specific area of the brain, causing a sudden loss of blood flow to the corresponding area of the brain and leading to neurological dysfunction [3]. Unlike in situ thrombosis in ACS, plaque ruptures in extracranial cervical arteries mostly result in distal embolization of the thrombus to the brain (arterial embolism), while the consequences of intracranial atherosclerotic plaque rupture are similar to those of ACS, namely bringing about in situ vessel occlusion [4]. Atherosclerosis and the build-up of plaque constrict blood vessels and reduce blood flow to the brain region, leading to severe stress and cell death due to hypoxia in the ischemic region.

In conclusion, thrombosis is the common pathogenetic process of myocardial infarction and ischemic stroke. Thus, thrombolysis plays a significant role in the treatment of these two diseases.

## Thrombolytic therapy

### The application of thrombolytic therapy in CVDs

Thrombolytic therapy, or thrombolysis, is to use the thrombolytic agents (TAs) to destroy or dissolve the thrombi in vessels. It is applied in various thrombotic or embolic CVDs, ranging from venous thromboembolism (VTE), acute ischemic stroke (AIS), acute myocardial infarction (AMI), to prosthetic valve thrombosis (PVT) [5].

As the mechanism of AMI and AIS is the acute artery occlusion which leads to ischemic necrosis of the tissue in its supplying area, to achieve recanalization and reperfusion as soon as possible is vital for avoiding irreversible damage and improving outcomes. Thrombolysis and interventional therapy, such as thrombectomy and stent implantation, are the two major approaches. For STEMI, though primary percutaneous coronary intervention (PCI) is a prior strategy [6, 7], it is hard to achieve, especially in regions with limited medical resources and emergency services, while it requires equipment for angiographic guidance and the evidence-based timeframe is restricted. When early PCI is not feasible, the application of thrombolysis before being transferred to facilities where catheterization is available provides an opportunity for early reperfusion at the symptom onset. The TRANSFER-AMI study has shown that, among the 1059 high-risk STEMI patients those who were treated with tenecteplase and then transferred for PCI within 6 h have a lower occurrence of endpoint composited of death, reinfarction, recurrent ischemia, new or worsening congestive heart failure, or cardiogenic shock within 30 days [8].

If PCI cannot be performed within the guideline-recommended timeframe, which is approximately 120 min, fibrinolytic drugs should be administered at full dose for patients under 75 years old, with the exclusion of contraindications [9]. The contraindications to thrombolytic therapy include uncontrolled hypertension, prior intracranial hemorrhage, history of head trauma within 3 months, intracranial surgery within 2 months, brain malignancy, cerebrovascular malformation, aortic dissection, active or recent bleeding, bleeding diatheses [5].

### Thrombolytic agents

Thrombolytic agents are natural or artificial substances that contribute to the fibrinolytic process, catalyzing the conversion of plasminogen to plasmin, which can degrade the essential component of thrombus, fibrin, into fibrin degradation products (FDPs). Features of several commonly used TAs are compared in Additional file 2 [10–25].

According to the time of discovery and characteristics, TAs are divided into 3 generations. In addition, TAs can be categorized as either “fibrin specific” or “non-fibrin specific”. The fibrin-specific TAs, including the second-and third-generation TAs, selectively activate the plasminogen that are bound to fibrin. Therefore, they pose a lower risk of complications attributed to systemic fibrinolytic activation. The first-generation TAs are streptokinase (SK) and urokinase (UK). The second-generation TAs, represented by recombinant tissue plasminogen activator (rt-PA) and single-chain urokinase (scu-PA), share common characteristics: They are to a certain extent fibrin specific but require large therapeutic dose and continuous intravenous infusion due to the short half-life. Among the new candidates of third-generation TAs, tenecteplase and reteplase are the two TAs approved by FDA for clinical treatment. They have a prolonged half-life, which allows them to be administered as a bolus dose rather than an infusion. Several clinical trials including RAPID II [18], PAPID II [19] and INJECT [25] have demonstrated that compared with other TAs such as rt-PA and SK, reteplase achieved a higher coronary artery patency rate without increasing the risk of bleeding or other adverse events.

The way of delivering TAs includes systematic delivery, Intracoronary (IC) thrombolysis, nanocarriers and so on. As a traditional way to deliver TAs, systematic delivery has the advantages of being convenient and affordable, while its limitations are non-specific bio-distribution and the risk of bleeding complications. IC thrombolysis is developed in 1990s as an adjunctive treatment to angioplasty, aimed at decreasing the risk of distal thromboembolism [26]. Through the direct administration of TAs in coronary artery, site-specific TA concentrations can be reached at high levels with fewer doses, and therefore posing less danger of systematic hemorrhage. Nanocarrier is a novel approach of drug delivery, which is still under clinical investigation [27, 28]. It conjugates with TAs, then under the trigger of an internal or external stimulus the conjugation disassembles at the thrombus site, thereby the concentration of TAs is increased.

## Risk prediction

### Clinical risks

As illustrated above, prompt treatment with fibrinolytic agents, such as alteplase, is an effective therapy for AIS [29] and STEMI [30], reducing mortality and improving recovery [30, 31]. However, it is associated with risks, which generally include in-hospital death (from any causes), recurrent occlusion, reperfusion injury, and immunologic and hemorrhagic complications.

Unsurprisingly, hemorrhage is the most common complication, since TAs may not only dissolve the protective blood clots, but lead to secondary hyperfibrinolysis, hypofibrinogenemia, platelet dysfunction, and other hemostatic defects [32] as well. Depending on site and severity, hemorrhagic complications can be further divided into several categories, among which intracranial hemorrhage (ICH) and major bleeding are most life-threatening and require specific treatment. The reported rates of post-thrombolysis ICH in AIS patients ranged from 0.2% to 1.0%, while the rate of major bleeding could reach 15% [33]. Since the diagnosis criteria might vary in different studies, these absolute rates are for reference only.

The allergic reactions are more often seen in patients given streptokinase as it is a heterologous protein. Acute anaphylaxis is severe but unusual, which may manifest as itching and redness of the skin, vasogenic edema, bronchospasm, dyspnea, hypotension, arrhythmia and shock.

Before applying thrombolytic therapy, in order to select the appropriate patient, reduce the risks of death or serious complications, and to get prepared in advance, it is necessary to carry out risk assessments and take the risk–benefit ratio into consideration.

### Risk scores

Clinical prediction scores for risk stratification and outcomes estimation of CVD patients have been developed in the past few decades. The regularly used risk scores are listed in Additional file 3 [34–43]. Though the scales for different CVDs vary, they have multiple indicators in common. Undoubtedly, the incidence of complications is correlated with the dose and type of TA applied. Besides, a variety of factors may play a role, including patient characteristics (age, gender, CVD history), symptom severity, comorbidities (hypertension on admission, diabetes mellitus, atrial fibrillation, coagulation defects), other treatments, etc. [29, 44].

Regarding ACS, the TIMI risk score and the GRACE score are regarded as the most universally used scales for ischemic risk stratification and prognosis prediction. Their prominent advantage is the easy bedside application attributed to the simple calculation method. There are two main versions of TIMI risk scores [34, 38] for STEMI and UA/NSTEMI respectively. They both have reliable identification of high-risk patients and excellent discriminatory power but are weak at generalization. Eagle KA et al. [37] designed the GRACE model, which has higher accuracy (c statistics 0.81) and is enabled to predict the in-hospital and 6-month mortality of the entire spectrum of ACS patients, including those with ST elevation or depression [45]. Hence GRACE can be easier generalized. Nevertheless, according to the original study, it is not applicable to patients being observed in an emergency department [37]. In contrast to the massive bleeding risk scores for PCI and antithrombotic therapy, those scores for thrombolysis are currently few.

When it comes to AIS, the NIHSS score [46], developed and validated by Thomas et al. in 1989, is used to determine stroke severity, treatment and prognosis [47]. This concise scale can be completed in 6.6 min, providing a quantitative measure of critical ingredients of a standard neurological examination [46, 48], and has become one of the predictors of post-thrombolysis ICH. The MSS score [40] is a simple clinical four-point risk score that combines age, NIHSS score, glucose and platelet count together. However, the original study only included 481 patients, compromising its validity. M Lou et al. [41] constructed the HAT score, a quick and easy-to-perform five-point scale considering the pretreatment NIHSS score, CT findings, DM history and blood glucose. Its limitations are that this score was developed in retrospective studies, the sample size was also inadequate. Consequently, the HAT score should be examined in larger cohorts and prospective studies before utilized in clinical decision making [41]. Compared with the HAT score, the SITS-ICH risk score [42] was based on a larger data set of 31,627 patients, and requires neither the measurement of blood platelet count (needed in the MSS score), nor the manifest infarct size on initial imaging (needed in the HAT score). Therefore, it can be more easily and immediately calculated. The DRAGON score [49] has a scoring similar to HAT score and uses the prestroke modified Rankin Scale (mRS) as one of the predicators, was originally developed to assess the short-term functional outcome, but has since been used to assess intracranial hemorrhagic risk. The initial study of GRASPS score [43] was the first to report that male sex and Asian race were independent risk factors. This well-validated score is an excellent clinical tool to assess the risk of intravenous tPA-related symptomatic ICH in patients treated with tPA within 3 h of stroke onset, but it cannot provide an indication on how much benefit patients would gain from this strategy. Likewise, none of these risk scores should be used as a justification of withholding thrombolytic therapy, because they are incapable of demonstrating the harm is greater than the benefit [40–43].

### Application of risk prediction in post-thrombolysis patients

Clinical studies are carried out to evaluate whether the scores or prediction models are reliable in risk prediction, here we sorted the studies conducted in recent years to give a clear and objective comparison on them (Table 1 [50–66] was shown at the end of the text). Studies which did not give an explicit statement that patients had received thrombolytic therapy were excluded.

**Table 1:** Accuracy of Risk Scores or Risk Prediction Models

First author, year	Risk score/ Risk prediction model	Data set	Observation period endpoint	Results
		Source size (n)		
Brewster LM, 2020 [50]	CKmax	TIMI 3B trial 1473	During hospitalization 1) Major bleeding 2) Composition of major bleeding, stroke and in-hospital death	1) AUC = 0.68 2) AUC = 0.69
	Age			1) AUC = 0.68 2) AUC = 0.67
	MV model (CK, age)			1) AUC = 0.80 2) AUC = 0.75
Chotechuang Y, 2020 [51]	GRACE	Primary data Low GRACE score (< 126 points) group: 229 Intermediate-high GRACE score (≥ 126 points) group: 112	6 months 1) Composite cardiovascular outcome 2) In-hospital mortality 3) Re-hospitalized with HF	1) AUC = 0.746; *p* = 0.003; HR = 5.02; OR = 5.69 2) *p* = 0.252 3) *p* < 0.001
Chotechuang Y, 2016 [52]	GRACE	Primary data Low GRACE score (< 126 points) group: 88 Intermediate-high GRACE score (≥ 126 points) group: 64	6 months 1) Composite cardiovascular outcome 2) In-hospital mortality 3) Re-hospitalized with HF 4) Cardiovascular death	1) AUC = 0.641; *p* = 0.024; HR = 2.97; OR = 3.20 2) *p* = 0.276 3) *p* = 0.036; OR = 5.34 4) AUC = 0.794
Hassan AKM, 2014 [53]	6MWTD	Primary data 100	3 months 1) MACE 2) HF 3) Re-infarction 4) Post-MI angina 5) Death	1) OR = 7.14; *p* < 0.001; 2) *p* = 0.001 3) *p* = 0.09 4) p: NS 5) *p* < 0.001
	GRACE			1) OR = 7.23; *p* = 0.004
	GRACE + 6MWT			1) OR = 8.14; *p* < 0.001
	TIMI			1) OR = 3.08; *p* = 0.07
Steyerberg EW, 2005 [54]	Belgium model	GUSTO-I 40,830	30 days All- cause mortality	AUC = 0.780
	TIMI-II			AUC = 0.782
	GISSI-II			AUC = 0.757
	GUSTO-I			AUC = 0.821
	GUSTO-I nomogram			AUC = 0.827
Nisar T, 2019 [55]	HAT	Primary data 89	During hospitalization SICH: NINDS, ECASS-II	AUC = 0.710, 0.769; *p* = 0.066, 0.044
	DRAGON			AUC = 0.786, 0.701; *p* = 0.012, 0.132
	SITS-ICH			AUC = 0.746, 0.655; *p* = 0.032, 0.247
	MSS			AUC = 0.730, 0.705; *p* = 0.044, 0.125
	SPAN-100			AUC = 0.547, 0.576; *p* = 0.681, 0.569
	SEDAN			AUC = 0.666, 0.617; *p* = 0.146, 0.383
	THRIVE			AUC = 0.543, 0.539; *p* = 0.688, 0.574
Asuzu D, 2015 [56]	DRAGON	Primary data 210	During hospitalization SICH: NINDS	AUC = 0.76
	s-TPI			AUC = 0.740
	ASTRAL			AUC = 0.72
	HAT			AUC = 0.70
	PRS			AUC = 0.66
	SEDAN			AUC = 0.66
	SITS-ICH			AUC = 0.65
	SPAN-100			AUC = 0.57
Watson-Fargie T, 2015 [57]	SEDAN	Primary data 431	During hospitalization SICH: NINDS, ECASS-II, SITS-MOST	AUC = 0.72, 0.67, 0.62
	HAT			AUC = 0.78, 0.73, 0.67
	GRASPS			AUC = 0.74, 0.69, 0.65
	SITS-ICH			AUC = 0.72, 0.72, 0.68
Van Hooff RJ, 2014 [58]	s-TPI	MISS and UZB 169	During hospitalization 1) Functional outcome: Excellent (mRS 0–1), Good (mRS 0–2), Catastrophic (mRS 5–6) 2)SICH: NINDS, ECASS II	1) AUC = 0.80, 0.83, 0.86
	iSCORE			1) AUC = 0.72, 0.80, 0.86
	DRAGON			1) AUC = 0.79, 0.82, 0.81
	MSS			2) AUC = 0.70, 0.86
	HAT			2) AUC = 0.67, 0.79
	SITS-SICH			2) AUC = 0.68, 0.76
	SEDAN			2) AUC = 0.70, 0.69
	GRASPS			2) AUC = 0.66, 0.83
Sung SF, 2013 [59]	MSS	Primary data 548	During hospitalization SICH: NINDS, ECASS II, SITS-MOST	AUC = 0.60, 0.62, 0.64
	HAT			AUC = 0.70, 0.69, 0.73
	SITS-ICH			AUC = 0.62, 0.61, 0.68
	GRASPS			AUC = 0.62, 0.61, 0.63
	SPAN-100			AUC = 0.56, 0.55, 0.57
Sung SF, 2013 [60]	SITS-ICH	Primary data ACS: 434 PCS: 84	During hospitalization SICH: NINDS, ECASS II, SITS-MOST, any ICH	ACS group: AUC = 0.64, 0.65, 0.70, 0.59
				PCS group: AUC = -, -, -, 0.79
Mazya M, 2013 [61]	SEDAN	SITS-ISTR SICH group: 2222 NO SICH group: 41,760	- SICH: NINDS, ECASS II, SITS-MOST, any ICH	SICH group: AUC = 0.64, 0.65, 0.70, 0.59
				NO SICH group: AUC = 0.79
Strbian D, 2014 [62]	MSS	Primary data 3012	During hospitalization SICH: NINDS, ECASS II, SITS-MOST, any ICH	AUC = 0.62, 0.63, 0.66, 0.63
	HAT			AUC = 0.65, 0.65, 0.64, 0.65
	SEDAN			AUC = 0.69, 0.70, 0.69, 0.70
	GRASPS			AUC = 0.67, 0.67, 0.67, 0.67
	SITS-ICH			AUC = 0.61, 0.64, 0.67, 0.64
	SPAN-100			AUC = 0.55, 0.56, 0.56, 0.56
Li M, 2015 [63]	SEDAN	TIMS-China 811	During hospitalization SICH: NINDS, ECASS II, SITS-MOST	AUC = 0.59, 0.59, 0.62
	SITS-ICH			AUC = 0.65, 0.69, 0.72
	GRASPS			AUC = 0.70, 0.73, 0.70
	MSS			AUC = 0.71, 0.72, 0.73
Chang X, 2021 [64]	ASPECTS	Primary data 248	During hospitalization Hemorrhagic transformation	AUC = 0.895; Se = 100%; Sp = 60.7%
	DRAGON			AUC = 0.877; Se = 84.4%; Sp = 82.1%
	SEDAN			AUC = 0.764; Se = 78.6%; Sp = 68.6%
	HAT			AUC = 0.777; Se = 68.8%; Sp = 82.1%
Orbán-Kálmándi R, 2021 [65]	CLA AUC	Primary data 231	90 days 1) SICH: ECASS II 2) Unfavorable functional outcomes (mRS > 2)/ no change	1) Se = 61.1%; Sp = 56.8% 2) Se = 49.5%; Sp = 66.7%
	Modified CLA (CLA in the presence of cfDNA and histones)			1) Se = 66.7%; Sp = 62.0% 2) Se = 64.2%; Sp = 55.2%
Turcato G, 2016 [66]	RDW	Primary data 316	1 year 1) Lack of neurological improvement 2) All-cause mortality	1) AUC = 0.667, *p* < 0.01 2) Median survival: 280 (RDW ≥ 14.5%) and 341(RDW < 14.5%) days

*DM* Diabetes melitus, *CKD* Chronic kidney disease, *DAPT* Dual antiplatelet therapy, *LMWH* Low molecular weight heparin, *AUC* Area under the receiver-operating characteristics curve, *OR* Odds ratio, *HR* Hazard ratio, *Se* Sensitivity, *Sp* Specificity, *TSOC ACS-DM* The Acute Coronary Syndrome-Diabetes Mellitus Registry of the Taiwan Society of Cardiology, *BRAVO* Building, Relating, Assessing, and Validating Outcomes, *EMPA-REG OUTCOME* Empagliflozin, Cardiovascular Outcomes, and Mortality in Type 2 Diabetes [67], *CANVAS* Canagliflozin Cardiovascular Assessment Study [68], *DECLARE-TIMI 58* Dapagliflozin and cardiovascular outcomes in type 2 diabetes [69], *MACE* Major adverse cardiovascular events, *6MWT* 6-min walk test, *ACSIS* Acute Coronary Syndrome Israeli Survey, *OTT* Onset to thrombolysis, *CREDO-Kyoto* Coronary Revascularization Demonstrating Outcome Study in Kyoto, *RESET* Randomized Evaluation of Sirolimus‐Eluting Versus Everolimus‐Eluting Stent Trial, *NEXT* Nobori Biolimus‐Eluting Versus Xience/Promus Everolimus‐Eluting Stent Trial, *ECASS-II* European-Australasian Cooperative Acute Stroke Study-II, *NINDS* National Institute of Neurological Disorders and Stroke, *SICH* symptomatic intracerebral hemorrhage, *CHF* Chronic heart failure, *AF* Atrial fibrillation, *MISS* Middelheim Interdisciplinary Stroke Study, *UZB* Universitair Ziekenhuis Brussel, *ACS* Anterior circulation stroke, *PCS* Posterior circulation stroke, *SITS-ISTR* Safe Implementation of Treatment in Stroke-International Stroke Thrombolysis Registry, *TIMS-China* Thrombolysis Implementation and Monitor of acute ischemic Stroke in China, *BUN/Cr* Blood urea nitrogen-to-creatinine ratio, *NLR* neutrophil-to-lymphocyte ratio, *DCA* Decision curve analysis, *NT-proBNP* N-terminal pro-brain natriuretic peptide, *CHD* coronary heart disease, *HT* Hemorrhagic transformation, *ASPECTS* Alberta stroke project early CT score, *CLA* clot lysis assay, *mRS* Modified RANKIN Sore, *RDW* Red blood cell distribution width

As for AMI, very few research discussed the performance of different risk scores in post-thrombolysis patients. Steyerberg EW et al. [54]  compared the accuracy of Belgium model, GISS-II, TIMI and GUSTO-I risk score for the prediction of the all-cause mortality at 30-day in 40,830 patients, GUSTO-I nomogram reached the highest AUC of 0.827. Besides, researchers have made attempts to discover new risk factors. Brewster LM et al. [50] applied a new multivariant model, with CK and age as predictors, to evaluate the risk of major bleeding and composite endpoint in 1473 patients, and the AUC reached 0.80 and 0.75, respectively, which was higher than other current scales. In addition, Hassan AKM et al. [53] combined GRACE and 6-min walk test in the evaluation of major adverse cardiovascular events (MACE), which gained a satisfying result with the OR of 8.14, higher than that of GRACE (OR = 7.03) and TIMI (OR = 3.08) alone. In general, GUSTO-I and GRACE risk scores performed better in the prediction of post-thrombolysis MACE. However, considering the lack of relevant studies and the potential bias between studies, a credible conclusion yet cannot be drawn. Compared with TIMI score, the scoring criteria of GUSTO-I and GRACE scores are more detailed, especially in the segmentation of age and heart rate. Despite the vital signs, the history of CVDs and some laboratory indicators are included as well. GUSTO-I score particularly takes ventricular function into consideration, by adding EF into the metrics. To further improve the accuracy of risk assessment, introducing more indicators seems to be a reasonable approach. Nevertheless, this is very likely to make the calculation more complicated, and the laboratory examination is time consuming, which is not feasible when an immediate risk assessment is in demand.

For post-thrombolysis AIS patients, one of the most life-threatening situations is hemorrhagic transformation (HT), which was often defined as symptomatic intracerebral hemorrhage (SICH). Traditional risk scores, including SEDAN, HAT, SITS-ICH, GRAPS, MSS, SPAN-100, and DRAGON, were utilized in HT prediction and their efficacy were validated by multiple clinical retrospective studies. Since each risk score was developed using different definitions to classify SICH, the variation of definitions across studies may have an impact on the accuracy. According to the initial study, the SEDAN and HAT scores used European Cooperative Acute Stroke Study II (ECASS II) definition, the GRASPS, MSS and SPAN-100 scores used the National Institute of Neurological Disorder and Stroke (NINDS) definition, while the SITS score used the SITS-MOST definition. Overall, the DRAGON score has a relatively higher predictive value, as its AUCs in different studies were all above 0.7 [55, 56, 64], with a median of 0.77. The HAT score also shows high reliability, whose AUC fluctuated between 0.64 and 0.78 [55–59, 62, 64]. In the research of Chang X et al. [64], which included 298 patients, the ASPECTS, DRAGON, HAT, and SEDAN scores achieved an AUC of 0.895, 0.877, 0.777, and 0.764, respectively. These scores all use the signs on admission CT scan as one of the scoring metrics, which may explain their better predictability. The SEDAN, MSS, SITS-ICH, and GRASPS scores had similar risk assessment capabilities, with the median AUC of 0.67, 0.68, 0.68, and 0.67, respectively. Among all the mentioned scores, SPAN-100 had the least satisfactory result. Sung SF et al. [59] applied SPAN-100 index in 548 patients and the AUC to predict SICH per NINDS, ECASS-II and SITS-MOST was only 0.56, 0.55 and 0.57, respectively.

Above all, thrombolysis risk scores or prediction models for AMI and AIS varied from each other in feature and accuracy. It is still hard to determine which one is the best in the complicated clinical conditions, especially with the inputs of multi-dimensional datatype and increasing data. Thus, more efficient and accurate approaches to make risk assessment is in need.

### Artificial Intelligence (AI) in risk prediction

Artificial intelligence (AI) refers to a branch of computer science that is developed to perform tasks that normally require human intelligence, perceiving environment and mimicking human cognitive behavior [70, 71]. Machine learning is one of the technical foundations of AI, which involves the automatic development of algorithms to identify patterns or groups in data [71]. When dealing with complex or massive data, higher accuracy can be achieved through machine learning over the traditional statistical methods. Deep learning, as a novel technique, uses multilayer neural networks to learn datasets with multiple levels of abstraction [72]. The representation of the input signal is learned by the network itself through training [73], and some deep learning models do not require manual supervision [74]. In this way, risks of systematic or random errors introduced by human factors are minimized. Deep convolutional neural networks (CNNs) and deep recurrent neural networks (RNNs) are two typical deep neural networks, specialized for specific learnings and can accomplish more complicated tasks through adequate combination [75]. With its high efficiency and accuracy, machine learning is nowadays increasingly applied in clinical processes, including diagnosis, treatment, prognosis and management of multiple diseases [76]. Good application prospect was also seen in the field of cardiocerebrovascular. Ambale V et al. [77] utilized random forest technique in the prediction of 6 cardiovascular events and reached higher prediction accuracy than other established risk scores. Johnson KM et al. [78]  used 5 machine learning methods to build models of vessel features, which better discriminated patients with subsequent adverse outcomes compared with conventional scores.

A number of researchers were devoted to the application of machine learning algorithms in post-thrombolysis risk prediction, identifying the potential predictors from various patient characteristics and developing new models (Table 2 [79–92] was shown at the end of the text).

**Table 2:** Accuracy of AI-based Risk Prediction Models

First author, year	Risk score/ prediction model/ predictors	Method	Dataset	Observation time endpoint	Results
			Source size		
Aziz F, 2021 [79]	New prediction models (Important variables: age, race, smoking status, hypertension, DM, family history of premature CVD, CRD, HR, BP, Killip class, blood glucose, administrations including cardiac catheterization, PCI, ASA, beta-blocker, ACEI, statins, diuretics, oral hypoglycemic agent and insulin)	RF	NCVD registry 12,368	1 year All-cause mortality: 1) In-hospital 2) at 30-day 3) at 1-year	1) AUC = 0.86; Se = 34.7%; Sp = 96.8%; Acc = 93.5% 2) AUC = 0.83; Se = 37.3%; Sp = 94.9%; Acc = 90.3% 3) AUC = 0.78; Se = 37.3%; Sp = 94.9%; Acc = 82.7%
		RFvarImp-SBE-RF			1) AUC = 0.87; Se = 33.7%; Sp = 97.7%; Acc = 94.2% 2) AUC = 0.85; Se = 41.3%; Sp = 93.0%; Acc = 88.9% 3) AUC = 0.80; Se = 46.0%; Sp = 90.1%; Acc = 83.8%
		RFE-RF			1) AUC = 0.86; Se = 34.7%; Sp = 96.4%; Acc = 93.1% 2) AUC = 0.82; Se = 38.7%; Sp = 95.2%; Acc = 90.7% 3) AUC = 0.79; Se = 49.2%; Sp = 88.9%; Acc = 83.2%
		SVM			1) AUC = 0.86; Se = 61.4%; Sp = 89.2%; Acc = 87.7% 2) AUC = 0.87; Se = 73.3%; Sp = 81.7%; Acc = 81.0% 3) AUC = 0.84; Se = 74.6%; Sp = 79.8%; Acc = 79.1%
		SVMvarImp-SBE-SVM			1) AUC = 0.88; Se = 69.3%; Sp = 86.1%; Acc = 85.2% 2) AUC = 0.90; Se = 84.0%; Sp = 79.0%; Acc = 79.4% 3) AUC = 0.84; Se = 75.4%; Sp = 77.3%; Acc = 77.1%
		RFE-SVM			1) AUC = 0.85; Se = 72.3%; Sp = 83.8%; Acc = 83.2% 2) AUC = 0.88; Se = 81.3%; Sp = 80.0%; Acc = 80.1% 3) AUC = 0.84; Se = 76.2%; Sp = 79.8%; Acc = 79.3%
		LR			1) AUC = 0.88; Se = 71.3%; Sp = 85.0%; Acc = 84.2% 2) AUC = 0.85; Se = 74.7%; Sp = 80.3%; Acc = 79.9% 3) AUC = 0.76; Se = 61.1%; Sp = 79.2%; Acc = 76.6%
		LRstepwise-SBE-LR			1) AUC = 0.89; Se = 75.2%; Sp = 84.1%; Acc = 83.6% 2) AUC = 0.85; Se = 74.7%; Sp = 83.4%; Acc = 82.7% 3) AUC = 0.80; Se = 65.9%; Sp = 81.4%; Acc = 79.2%
		RFE-LR			1) AUC = 0.87; Se = 66.3%; Sp = 83.4%; Acc = 82.5% 2) AUC = 0.83; Se = 72.0%; Sp = 81.0%; Acc = 80.3% 3) AUC = 0.78; Se = 61.9%; Sp = 80.2%; Acc = 77.6%
	TIMI	-			1) AUC = 0.81; Se = 64.4%; Sp = 83.4%; Acc = 82.4% 2) AUC = 0.80; Se = 62.7%; Sp = 83.2%; Acc = 81.6% 3) AUC = 0.76; Se = 48.4%; Sp = 83.7%; Acc = 78.6%
Xu Y, 2022 [80]	New prediction models (Important variables: triglycerides, Lpa, baseline NIHSS score, hemoglobin, BP, INR, WBC, etc.)	RF	Primary data 345	- Hemorrhagic transformation	AUC = 0.795; Se = 66.7%; Sp = 80.7%
		LR			AUC = 0.703; Se = 60.0%; Sp = 78.0%
	SITS-ICH	-			AUC = 0.660
	MSS	-			AUC = 0.657
	SEDAN	-			AUC = 0.655
Meng Y, 2022 [81]	New clinical model	RF	Primary data 71	- Hemorrhagic transformation	AUC = 0.556; Se = 33.3%; Sp = 55.6%; Acc = 54.5%; F1 score = 0.067
	New radiomics models				Abnormal ROIs: AUC = 0.831; Se = 60.0%; Sp = 88.2%; Acc = 81.8%; F1 score = 0.600 All ROIs: AUC = 0.871; Se = 73.3%; Sp = 88.2%; Acc = 84.8%; F1 score = 0.687
	Combined model				AUC = 0.911; Se = 81.0%; Sp = 93.3%; Acc = 89.4%; F1 score = 0.830
Weng ZA, 2022 [82]	New nomogram (Smoking, NIHSS, BUN/Cr, and NLR)	LR	Primary data Training group: 387 Testing group: 166	During hospitalization Any ICH	Training group: AUC = 0.887; calibration plots mean absolute error = 0.025; DCA threshold probabilities = 2.5–57.8% Testing group: AUC = 0.776; calibration plots mean absolute error = 0.036; DCA threshold probabilities = 5.4–38.2%
	MSS	-			Training group: AUC = 0.723 Testing group: AUC = 0.647
	GRASPS	-			Training group: AUC = 0.738 Testing group: AUC = 0.671
	SPAN-100	-			Training group: AUC = 0.538 Testing group: AUC = 0.552
Zhang K, 2022 [83]	New nomogram (DM, AF, total cholesterol, fibrous protein, cerebral infarction area, NIHSS score, onset-to-treatment)	LR	Primary data Training group: 392 Testing group: 178	During hospitalization SICH: ECASS II	Training group: AUC = 0.831 Testing group: AUC = 0.889; Sp = 82.3%; Se = 83.3% Hosmer–Lemeshow test calibration = 7.466
Zhang KJ, 2021 [84]	NT-proBNP	-	Primary data 404	3 months 1) Hemorrhagic transformation 2) 3-month mortality	1) *p* < 0.001; OR = 1.341 2) *p* < 0.001; OR = 1.788
	New nomogram (NT-proBNP, NIHSS scores and baseline glucose levels)	LR	Primary data Training group: 283 Testing group: 121	3 months Poor 3-month functional outcomes	Training group: AUC = 0.710; Se = 72.3%; Sp = 60.8% Testing group: AUC = 0.764; Se = 80.3%; Sp = 64.9%
Guo H, 2021 [85]	New nomogram (AF, baseline glucose level, NLR, and baseline NIHSS)	LR	Primary data 1200	During hospitalization SICH: ECASS II	AUC = 0.788
Soni M, 2021 [86]	New risk score (Age > 75 years, BP, severity of stroke, pre-treatment antithrombotic and history of hypertension and hyperlipidemia)	K-means clustering	Primary data 890	During hospitalization SICH: ECASS II	AUC = 0.75 (continuous risk scoring), 0.71 (risk stratification)
Liu J, 2021 [87]	New radiomic model	LASSO regression	Primary data 109	90 days 1) Hemorrhage expansion (a 33% increase in the hematoma volume) 2) 3-month mortality 3) 3-month mortality/ disability (mRS score 3–6)	1) AUC = 0.85; Se = 82%; Sp = 77%; Acc = 78% 2) AUC = 0.67; OR = 5.17 3) AUC = 0.63; OR = 4.70
			After SMOTE data Training group: 96 Testing group: 23		Training group: 1) AUC = 0.91; Se = 83%; Sp = 89%; Acc = 87% Testing group: 1) AUC = 0.87; Se = 60%; Sp = 85%; Acc = 74%
Chen Z, 2021 [88]	New radiomic models	LR	Primary data 40	- 1) No recanalization 2) Full recanalization	1) AUC = 0.751; MCC = 0.730 2) AUC = 0.797; MCC = 0.762
		ALEM			1) AUC = 0.804; MCC = 765 2) AUC = 0.866; MCC = 0.841
		CNN			1) AUC = 0.781; MCC = 0.758 2) AUC = 0.814; MCC = 792
		U-NL-NET			1) AUC = 0.844; MCC = 0.827 2) AUC = 0.875; MCC = 0.853
		AUNet			1) AUC = 0.875; MCC = 0.851 2) AUC = 0.898; MCC = 0.863
Wang F, 2020 [89]	New prediction models (Important variables: age, AF, glucose level, NIHSS score, and DNT)	SVM	Primary data 2237	- SICH	AUC = 0.79
		Neural Network			AUC = 0.82
		LR			AUC = 0.77
		AdaBoost			AUC = 0.77
		RF			AUC = 0.76
Bacchi S, 2020 [90]	New prediction models	CNN + ANN CTB + clinical data	Primary data 204	90 days 1) 24-h outcome (based on NIHSS) 2) 90-day outcome (based on mRS)	1) AUC = 0.70; Se = 93%; Sp = 53%; Acc = 71% 2) AUC = 0.75; Se = 56%; Sp = 93%; Acc = 74%
		ANN			1) AUC = 0.68; Se = 43%; Sp = 88%; Acc = 68% 2) AUC = 0.61; Se = 81%; Sp = 47%; Acc = 65%
		CNN CTB			1) AUC = 0.63; Se = 71%; Sp = 65%; Acc = 68% 2) AUC = 0.54; Se = 88%; Sp = 40%; Acc = 65%
	THRIVE	-			1) AUC = 0.63; Se = 43%; Sp = 76%; Acc = 61% 2) AUC = 0.69; Se = 81%; Sp = 47%; Acc = 65%
	HAT	-			1) AUC = 0.67; Se = 71%; Sp = 59%; Acc = 65% 2) AUC = 0.63; Se = 56%; Sp = 67%; Acc = 61%
	SPAN 100	-			1) AUC = N/A; Se = 21%; Sp = 88%; Acc = 58% 2) AUC = N/A; Se = 100%; Sp = 33%; Acc = 67%
Chung CC, 2020 [91]	New prediction models (Important variables: BP, HR, glucose level, consciousness level, NIHSS score, and history of DM)	ANN	Primary data Training group: 157 Testing group: 39	24 h major neurologic improvement (based on mRS)	Training group: AUC = 0.950; Acc = 97.5% Testing group: AUC = 0.944; Se = 89.8%; Sp = 95.9%; Acc = 94.6%
		ANN		3-month outcome (based on mRS)	Training group: AUC = 0.992; Acc = 98.2% Testing group: AUC = 0.933; Se = 94.3%; Sp = 86.5%; Acc = 88.8%
Bentley P, 2014 [92]	New radiomic models	Automated SVM	Primary data 116	During hospitalization SICH: NINDS	AUC = 0.744
		Manual SVM			AUC = 0.671
	SEDAN	-			AUC = 0.626
	HAT	-			AUC = 0.629
	SEDAN-NIHSS, CT only	-			AUC = 0.720
	HAT-NIHSS, CT only	-			AUC = 0.648

*DM* Diabetes mellitus, *CRD* Chronic renal disease, *HR* Heart rate, *BP* Blood pressure, *RF* Random Forest, *RFvarImp-SBE-RF* RF variable importance with sequential backward elimination and RF classifier, *RFE-RF* Recursive feature elimination with RF classifier, *SVM* Support Vector Machine, *SVMvarImp-SBE-SVM* SVM variable importance with sequential backward elimination and SVM classifier, *RFE-SVM* Recursive feature elimination with SVM classifier, *LR* Logistic Regression, *LRstepwise-SBE-LR* LR stepwise feature elimination and LR classifier, *RFE- LR* Recursive feature elimination with LR classifier, *PCI* Percutaneous coronary intervention, *ASA* Acetylsalicylic acid (aspirin), *ACEI* Angiotensin-converting enzyme inhibitor, *NCVD* National Cardiovascular Database, *NLR* neutrophil-to-lymphocyte ratio, *AF* Atrial fibrillation, *AdaBoost* Adaptive boosting, *ADT* Alternating decision tree, *PART* Pruning rules based classification tree, *ACSIS* Acute Coronary Syndrome Israeli Survey, *PAT* Previous antiplatelet therapy, *FRT* Futile recanalization therapies, *ML* Machine learning, *IVT* Intraarterial thrombolysis, *mRS* Modified Rankin Scale, *ROIs* Regions of interest, *Acc* Accuracy, *LR* Logistic regression method, *LASSO* Least absolute shrinkage and selection operator, *CNN* Convolutional neural network, *ALEM* Adaptive linear ensemble model, *DNT* Door-to-needle time, *U-NL-NET* U-Net network with an accelerated non-local module, *MCC* Matthew’s correlation coefficient, *SVM* Support vector machines, *MCA* Middle cerebral artery, *ANN* Artificial neural networks, *CTB* Noncontrast CT brain, *HIAT* Houston Intra-Arterial Therapy score, *SMOTE* Synthetic minority oversampling technique

The effectiveness of these models was validated through the comparison with traditional scores. In several studies, new nomograms were generated through logistic regression analysis, among which that developed by Zhang K et al. [83] reached a high AUC of 0.889 when predicting the risk of HT in 178 patients. Aziz F et al. [79] applied random forest, support vector machine and logistic regression models to predict short- and long-term mortality among heterogenous Asian STEMI patients. AUCs from 0.73 to 0.90 are achieved, with the highest AUCs of 0.89, 0.90 and 0.84 for hospitalization, 30 days, and 1 year respectively, outperforming TIMI risk score whose AUCs are 0.81,0.80 and 0.76. When it comes to AIS, in most studies AI models showed better prediction ability of hemorrhagic complications than traditional risk scores or statistically based models. Some researchers extracted radiomic features and utilized machine learning to build radiomics models. Meng Y et al. [81]  extracted 5,400 radiomic features from 20 normal and abnormal regions of interest (ROIs) of MRI images among 71 patients, used the least absolute shrinkage and selection operator (LASSO) regression for feature selection, and constructed a radiomics model through RF, which was combined with 16 screened clinical factors with better support. The AUC with All-ROIs reached 0.871 and was further promoted to 0.91 when combined with other clinical factors. In addition to HT prediction after thrombolysis, radiomics-based models can also evaluate hemorrhage expansion well. Liu J et al. [87]  applied LASSO regression to identify five optimal radiomic image features on non-contrast-enhanced CT (NECT) as predictors, and developed a quantitative radiological score with a maximum AUC of 0.91. Besides, some researchers have also utilized deep learning to construct thrombolytic risk assessment models for AIS patients. To predict 24-h and 90-day functional outcomes better, Bacchi S et al. [90]  constructed a new prediction model among 204 stroke patients, using CNN and artificial neural networks (ANN), and found that a combination of CNN and ANN based on CT image and clinical data had the best performance, with the highest AUC of 0.70 and 0.75, respectively. Wang F et al. [89] have used logistic regression (LR), neutral network, support-vector machine (SVM), random forest (RF) and adaptive boosting (AdaBoost) to develop five machine learning models in 2237 cases for post-thrombolysis sICH prediction, screening out the five most valuable input factors (age, AF, glucose, NIHSS score and door-to-need time). The three-layer neural network model performed best and its AUC was 0.82. Among 40 patients, Chen Z et al. [88] have proposed a new prediction model named AUNet, which combined the features of an adaptive linear ensemble model (ALEM) and a deep U-Net network with an accelerated non-local module (U-NL-Net), to predict infarct volumes for AIS patients with or without recanalization, and the AUCs were 0.898 and 0.875, respectively.

AI models have obvious advantages in post-thrombolysis risk prediction, including high efficiency, higher accuracy when dealing with massive and multi-dimensional data, the capacity of comparing different methodologies on the same database, suitability for multi-ethnic population and so on. Undeniably, there are certain shortcomings, such as poor interpretability, weak generalization, and the unsatisfactory accuracy of some models. To solve these problems, efforts should be made in the improvement of algorithms, the enhancement of interpretability analysis, and the establish of multi-centered and normalized databases.

## Conclusion

Among CVDs, blockage diseases such as CHD and stroke are the leading cause of death, imposing a huge burden to the public health. The key treatment is to recanalize the embolized vascular and restore blood supply in the ischemic area. Thrombolytic therapy is a basic therapy in the recanalization strategy, whose advantages lays in rapidity, economy and non-invasiveness. However, the thrombolysis-related clinical risks such as hemorrhagic complications, futile recanalization and reocclusion, restrict the use of thrombolysis, yet make the pretreatment risk assessment necessary. New risk scores and AI-based prediction models are therefore continually developed and modified. It is still hard to say which risk score can achieve the highest accuracy, but with the continuous improvement of risk prediction, the application of thrombolysis will be relatively safer, which definitely brings great benefits to patients.

### Supplementary Information

**Additional file 1. **The Pathogenic Mechanism of Atherosclerosis.

**Additional file 2. **Features of Different TAs.

**Additional file 3. **Traditional Risk Scores for ACS and AIS.

## Data Availability

Not applicable.
