# Supplementary figures and images for: Application and risk prediction of thrombolytic therapy in cardio-cerebrovascular diseases: a review
Source: Thromb J. 2023 Sep 4;21:90. doi: 10.1186/s12959-023-00532-0 (PMC10476453; doi:10.1186/s12959-023-00532-0)

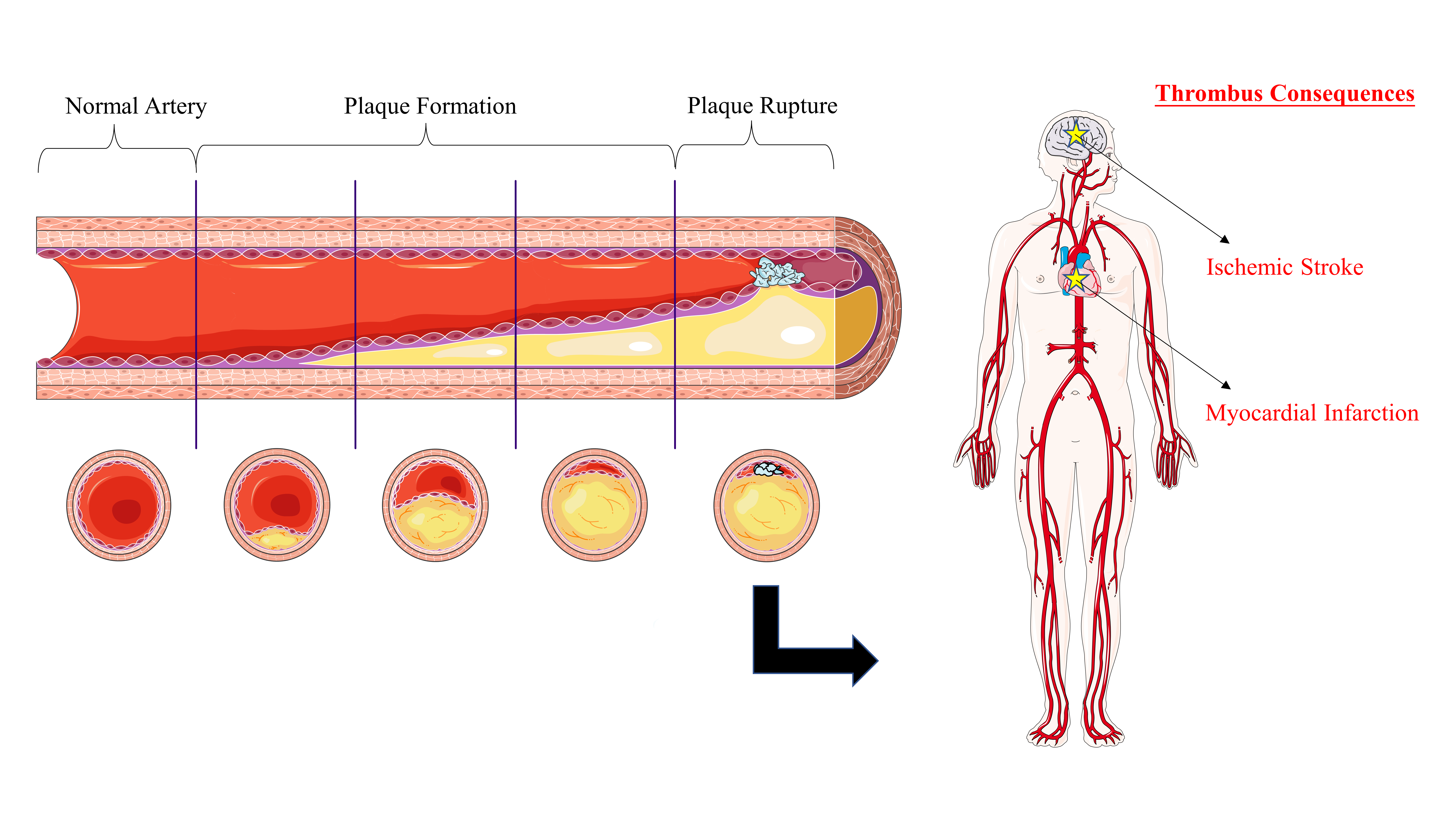

Supplement: Supplementary file 1 — Additional file 1. The Pathogenic Mechanism of Atherosclerosis. [file 12959_2023_532_MOESM1_ESM.tif]
