# Supplementary material for: Application and risk prediction of thrombolytic therapy in cardio-cerebrovascular diseases: a review
Source: Thromb J. 2023 Sep 4;21:90. doi: 10.1186/s12959-023-00532-0 (PMC10476453; doi:10.1186/s12959-023-00532-0)
Supplement: Supplementary file 2 — Additional file 2. Features of Different TAs. [file 12959_2023_532_MOESM2_ESM.docx]

**Additional file 2. Features of Different TAs**

| Thrombolytic agents | | | Streptokinase | Urokinase | Alteplase | Saruplase | Tenecteplase | Reteplase |
| --- | --- | --- | --- | --- | --- | --- | --- | --- |
| Abbreviation | | | SK | UK | rt-PA | scu-PA | TNK-tPA | r-PA |
| Indication | | | AMI, PE, DVT, PAO | AMI, PE, PAO, PVT | AMI, AIS, PE, PAO, PVT | AMI | AMI, PE | AMI |
| Source | | | β-hemolytic streptococcus | Human urine | Bioengineering | Human urine | Bioengineering | Bioengineering |
| Immunogenicity | | | + | - | - | - | - | - |
| Fibrin-specificity | | | - | - | ++ | +++ | +++ | + |
| Half-life (min) | | | 18 | 8-20 | 4-8 | 7-10 | 10-24 | 14-19 |
| AMI | Dose | | 1.5MU | 1.5MU+1.5MU | 15mg+85mg | 20mg+60mg | 30-50mg | 10MU+10MU |
|  | Administration | | 60 min infusion | Bolus+  90 min infusion | Bolus+  90 min infusion | Bolus+  60 min infusion | Single bolus | Double bolus |
|  | TIMI 3 flow rate at 90 min (%) | | 32 | 40-54 | 45-68 | 53-72 | 54-66 | 60-63 |
|  | Clinical  outcomes | Reinfarction (%) | 4.5 | 2.0-4.0 | 4.2-5.7 | 4.2-5.4 | 2.6-5.2 | 4.2 |
|  |  | Stroke (%) | 1.0-1.3 | 2.0-3.0 | 0.8-1.9 | 0.5-1.4 | 1.0-3.8 | 1.2-1.6 |
| AIS | Dose | | - | - | 0.9mg/kg  10%+90% | - | - | - |
|  | Administration | | - | - | Bolus+  60 min infusion | - | - | - |

AMI: acute myocardial infarction, PE: pulmonary embolism, DVT: deep venous thrombosis, PAO: peripheral arterial occlusion, PVT: prosthetic valve thrombosis, AIS: acute ischemic stroke.
