# Supplementary material for: Application and risk prediction of thrombolytic therapy in cardio-cerebrovascular diseases: a review
Source: Thromb J. 2023 Sep 4;21:90. doi: 10.1186/s12959-023-00532-0 (PMC10476453; doi:10.1186/s12959-023-00532-0)
Supplement: Supplementary file 3 — Additional file 3. Traditional Risk Scores for ACS and AIS. [file 12959_2023_532_MOESM3_ESM.docx]

Additional file 3

**Table 1(a). Traditional Risk Scores for ACS**

| Risk score | Author | Disease | Sample size | Scoring Metrics | | | | | | | | | | | Endpoint | Risk Stratification |
| --- | --- | --- | --- | --- | --- | --- | --- | --- | --- | --- | --- | --- | --- | --- | --- | --- |
|  |  |  |  | Age/y | SBP/mm Hg | HR/bpm | Weight/kg | Comorbidities | History | Killip | ECG | Laboratory Metrics | Time to Rx/h | Remark |  |  |
| TIMI Risk Score | Morrow D et al, 2000 | STEMI | 14144 | 65-74 (2)  ≥75 (3) | <100 (3) | >100 (2) | <67 | DM/HTN/angina (1) | - | II-IV (2) | Anterior STE or LBBB (1) | - | >4 | - | All-cause mortality within 30 days. | Low: 0-3;  Intermediate: 4-5  High: >5 |
| Dynamic TIMI Risk Score | Amin S et al, 2013 | STEMI | 22335 | 65-74 (2)  ≥75 (3) | <100 (3) | >100 (2) | <67 (1) | DM/HTN/angina (1) | - | II-IV (2) | Anterior STE or LBBB (1) | - | >4 (1) | Recurrent MI (1); Stroke (5); Major bleeding (1); CHF/shock (3); Arrhythmia (2); Renal failure (3). | Death, nonfatal MI, or nonfatal stroke. | Low: 0/1;  Moderate: 4;  High: ≥8. |
| GUSTO-I score (without angiographic data) | Callif R et al,  2000 | STEMI | 41021 | 30 (10)  40 (15)  50 (20)  60 (32)  70 (46)  80 (59)  90 (73)  100 (86) | - | - | - | - | Previous infarction (18) | - | - | - | - | In-hospital CHF/PE (25) | Death. |  |
| GUSTO-I score (with angiographic data) |  |  |  | 30 (1)  40 (1)  50 (2)  60 (3)  70 (5)  80 (6)  90 (8) | - | 40 (0)  60 (1)  80 (2)  100 (3)  ≥120 (4) | - | - | Previous infarction (2) | - | - | - | - | EF /%: 10 (99); 20 (98); 30 (97); 40 (96); 50 (95); 60 (94); 70 (93); 80 (92).  In-hospital CHF/PE (2) |  |  |
| GRACE | Eagle K et al,  2004 | ACS | 22645 (development cohort: 15007; validation cohort: 7638) | ≤29 (0)  30-39 (0)  40-49 (18)  50-59 (36)  60-69 (55)  70-79 (73)  80-89 (91)  ≥90 (100) | ≤79.9 (24);  80-99.9 (22);  100-119.9 (18);  120-139.9 (14);  140-159.9 (10);  160-199.9 (4);  ≥200 (0) | ≤49.9 (0);  50-69.9 (3);  70-89.9 (9);  90-109.9 (14);  110-149.9 (23);  150-199.9 (35);  ≥200 (43) | - | - | CHF (24);  MI (12) | - | ST-segment depression (11) | Initial Scr, mg/dl:  0-0.39 (1);  0.4-0.79 (3)  0.8-1.19 (5)  1.2-1.59 (7)  1.6-1.99 (9)  2-3.99 (15)  ≥4 (20)  Elevated Cardiac Enzymes (15) | - | No In-Hospital PCI (14). | All-cause mortality. |  |
| TIMI Risk Score | Antman E et al, 2000 | UA/NSTEMI | 7081  (TIMI 11B: n=3910, ESSENCE: n= 3171) | ≥65 (1) | - | - | - | - | - | - | ST deviation (1) | Serum cardiac markers elevation (1) | - | ≥3 risk factors for CAD (1); significant coronary stenosis (1); severe anginal symptoms (1); aspirin use in last 7 days (1). | A composite of all-cause mortality, MI, or severe recurrent ischemia. | Low: 0-1;  Indeterminate: 2;  Intermediate: 3-4;  High: 5-7. |
| CRUSADE bleeding score | Subherwal S et al, 2009 | NSTEMI | 71277 | - | ≤90 (10)  91-100 (8)  101-120 (5)  121-180 (1)  181-200 (3)  ≥201 (5) | ≤70 (0)  71-80 (1)  81-90 (3)  91-100 (6)  101-110 (8)  111-120 (10)  ≥121 (11) | - | DM (6) | Vascular disease (6) | - | - | Baseline hematocrit, %:  <31 (9)  31-33.9 (7)  34-36.9 (3)  37-39.9 (2)  ≥40 (0) | - | CHF signs at presentation (7)  Gender: male (0); female (8).  Creatinine clearance, mL/min:  ≤15 (39)  >15-30 (35)  >30-60 (28)  >60-90 (17)  >90-120 (7)  >120 (0) | Major bleeding. | Very low: ≤20;  Low: 21-30;  Moderate: 31-40;  High: 41-50;  Very high: >50. |

SBP: systolic blood pressure, HR: heart rate, ECG: electrocardiogram, rx: treatment, TIMI: Thrombolysis in Myocardial Infarctio, STEMI: ST-segment elevation myocardial infarction, DM: diabetes mellitus, HTN: hypertension, STE: ST-segment elevation, LBBB: Left Bundle Branch Block, MI: myocardial infarction, CHF: congestive heart failure, GUSTO-I: the Global Utilization of Streptokinase and TPA (alteplase) for Occluded Coronary Arteries, PE: pulmonary embolism, EF: ejection fraction, GRACE: Global Registry of Acute Coronary Events, ACS: acute coronary syndrome, Scr: Serum Creatinine, PCI: Percutaneous Coronary Intervention

**Table 1(b). Traditional Risk Scores for AIS**

| Risk score | Author | Sample size | Scoring Metrics | | | | | | | | | Endpoint | Risk Stratification |
| --- | --- | --- | --- | --- | --- | --- | --- | --- | --- | --- | --- | --- | --- |
|  |  |  | Age/y | Gender | SBP/mm Hg | History | Medication use | Baseline NIHSS | Laboratory Metrics | OTT/min | Remark |  |  |
| HAT score | Lou M et al, 2008 | 609 | - | - | - | DM or glucose> 200 mg/dL (1) | - | <15 (0)  15-20 (1)  ≥20 (2) | - | - | Hypodensity presence on initial head CT scan:  No (0)  <1/3 of MCA territory (1)  ≥1/3 of MCA territory (2) | ICH. | Higher risk: >3;  Low risk: ≤2. |
| MSS score | Cucchiara B et al, 2008 | 481 | >60 (1) | - | - | - | - | >10 | Glucose, mg/dL:  >150 (1);  Platelet count/mm^3^:  <150,000 (1) | - | - | Symptomatic ICH;  Asymptomatic ICH;  Parenchymal hemorrhage (PH). | ICH rate:  0: 2.6%;  1: 9.7%;  2: 15.1%;  ≥3: 37.9%. |
| SITS-SICH risk score | Mazya M et al, 2012 | 31627 | ≥72 (1) |  | ≥146 (1) | Hypertension (1) | Aspirin + clopidogrel (3)  Aspirin monotherapy (2) | ≥13 (2)  7-12 (1) | Glucose, mg/dL:  ≥180 (2) | ≥180 (1) | Weight≥95 kg (1) | SICH per the SITS-MOST definition. | Low: 0-2;  Average: 3-5;  Moderate: 6-8;  High: ≥9. |
| GRASPS score | Menon B et al, 2012 | 10242 | ≤60 (8)  61-70 (11)  71-80 (15)  >80 (17) | Male (4)  Female (0) | <120 (10)  120-149 (14)  150-179 (18)  ≥180 (21) | - | - | 0-5 (25)  6-10 (27)  11-15 (34)  16-20 (40)  >20 (42) | B-Glucose, mg/dL:  <100 (2)  100-149 (6)  ≥150 (8) | - | Ethnicity:  Asian (9)  Non-Asian (0) | ICH | tPA-related ICH rate:  45: ≤1%;  60: 2%;  80: 8%;  100: 28% |

NIHSS: National Institutes of Health Stroke Scale, OTT: onset-to-treatment, HAT score: hemorrhage after thrombolysis score, MCA: middle cerebral artery, ICH: intracranial hemorrhage, SITS SICH risk score: Safe Implementation of Treatments in Stroke Symptomatic Intracerebral Hemorrhage Risk Score, SITS-MOST: Safe Implementation of Thrombolysis in Stroke-Monitoring Study, GRASPS: Glucose at presentation, Race (Asian), Age, Sex (male), systolic blood Pressure at presentation, and Severity of stroke at presentation (NIHSS), B-Glucose: Blood-Glucose
